# Adsorption Behavior and Mechanism of Cesium Ions in Low-Concentration Brine Using Ammonium Molybdophosphate–Zirconium Phosphate on Polyurethane Sponge

**DOI:** 10.3390/ma16134583

**Published:** 2023-06-25

**Authors:** Hao Wang, Guihua Ma, Ke Zhang, Zhi Jia, Yuzhuo Wang, Li Gao, Bingxin Liu

**Affiliations:** School of Mechanical Engineering, Qinghai University, Xining 810016, China; wanghao1512@sinap.ac.cn (H.W.); mgh1801263258@126.com (G.M.); 15092385877@163.com (K.Z.); jz17852918128@163.com (Z.J.); wyz20030806@163.com (Y.W.)

**Keywords:** Cs^+^ adsorption, polyurethane sponge, ammonium molybdophosphate, zirconium phosphate

## Abstract

Salt lake brine originating from Qinghai, China has abundant cesium resources and huge total reserves. The inorganic ion exchangers ammonium molybdophosphate (AMP) and zirconium phosphate (ZrP) have the significant advantages of separating and extracting Cs^+^ as a special adsorbent. Nevertheless, their high solubility in water leads to a decrease in their ability to adsorb Cs^+^ in aqueous solutions, causing problems such as difficulty with using adsorbents alone and a difficult recovery. In this work, an environmentally friendly polyurethane sponge (PU sponge) with a large specific surface area is employed as an adsorbent carrier by physically impregnating dopamine-coated AMP and ZrP onto a PU sponge, respectively. The experiment found that under the same conditions, the AMP/PU sponge performs better than the ZrP/PU sponge for Cs^+^ adsorption. When the amount of adsorbent reaches 0.025 g, the adsorption capacity reaches saturation. The adsorption efficiency remains above 80% when the concentration of Cs^+^ is 5–35 mg/L. The kinetic calculations show that adsorption is spontaneous, feasible, and has a higher driving force at high temperatures. In addition, the power and mechanism of the interaction between adsorbent and adsorbent are explained using the density functional theory calculation. This efficient, stable, and selective Cs^+^ adsorbent provides design guidelines.

## 1. Introduction

Because of their excellent physical and chemical properties and outstanding photosensitivity, cesium and its compounds have received increasing attention all over the world. Cesium has a wide range of applications, including chemical engineering, electronics, aerospace, medicine, and so on [1,2,3]. The salt lake brine in Qinghai China is a source of abundant cesium resources and gigantic total reserves [4,5,6]. However, it has the characteristics of a low concentration and complex chemical environments. This poses a great challenge to the utilization and enrichment of cesium resources in salt lake brine. Adsorbents are momentous carriers used to accelerate the enrichment, separation, and extraction of cesium ions, and they play a catalytic role in the utilization and collection of cesium resources. 

Currently, precipitation, solvent extraction, and ion exchange are approaches used to separate and extract Cs^+^ from salt lake brine [7,8,9]. Of these, ion exchange is considered to be one of the most promising methods for the separation and extraction of Cs^+^ in salt lake brine due to its simple process and environmental friendliness [10]. Ammonium molybdophosphate ((NH_4_)_3_PMo_12_O_40_·xH_2_O, AMP) and zirconium phosphate (Zr(HPO_4_)_2_·2H_2_O, ZrP) are widely investigated special-effect adsorbents of Cs^+^ [11]. Nevertheless, it is difficult for AMP and ZrP to directly and efficiently separate and extract Cs^+^ as an adsorbent because of their microcrystalline structures, small specific surface areas, poor mechanical strengths, and easy solubility in water. To this end, various inorganic materials are often used as carriers of AMP and ZrP. Xia [12] synthesized an Fe_3_O_4_-CTS-AMP composite of Fe_3_O_4_-chitosan particles combined with ammonium molybdophosphate using a facile method through a coordination with CTS and AMP, and the maximum adsorption capacity was calculated as 177.8 mg/g. Chen [13] prepared a composite hydrogel (APS) of ammonium molybdophosphate (AMP)/polyvinyl alcohol (PVA)/sodium alginate (SA) for Cs^+^ removal and enrichment from radioactive waste water. This APS has a high permeability, and a maximum adsorption capacity of 71.28 mg/g, which can be achieved within a short time. Ding [14] prepared an ammonium molybdophosphate-polyacrylonitrile (AMP-PAN) membrane which can efficiently remove Cs^+^ (95.7%, 94.1%, and 91.3% of 1 mg/L) from solutions with high ionic strength (400 mg/L of Na^+^, Ca^2+^ or K^+^), and the estimated maximum adsorption capacities even reached 138.9 ± 21.3 mg/g. Jiao [15] prepared a composite of zirconium phosphate (ZrP) loaded on mesoporous silica pellets (MSP) via the one-pot liquid phase grafting method to develop an effective inorganic adsorbent for strontium (Sr) ion removal from contaminated water. The synthesized composite (i.e., ZrP/MSP) exhibited excellent performance for Sr^2+^ adsorption over a wide pH range and achieved a much faster adsorption equilibrium (90 min) and a higher maximum adsorption capacity (100.77 mg/g) than the bulk ZrP (i.e., 180 min for 68.88 mg/g) without grafting. Abdel Maksoud [16] synthesized a zinc ferrite and humic acid (ZFO/HA) nanocomposite material for the effective adsorption of cesium. Its monolayer capacity is 42.55 mg/g, and it was found that the adsorption reaction is a spontaneous endothermic reaction. Li [17] synthesized two types of dicalcium magnesium phosphate with significant adsorption capacities for Cs^+^, KMgPO_4_ H_2_O (KMP), and NH_4_MgPO_4_·H_2_O (NMP), which were the highest among all reported adsorbents, accounting for 84% of their theoretical values. Ye [18] prepared a composite spherical adsorbent using ammonium molybdate phosphate (AMP), sodium alginate, and calcium chloride as raw materials. It was found that the adsorption rates of the porous adsorbent for rubidium and cesium were extremely fast. Nevertheless, its high solubility in water leads to a decrease in its ability to adsorb Cs^+^ in aqueous solutions and causes problems such as difficulty with using the adsorbent alone and a difficult recovery.

Based on previous studies, it was found that the inorganic ion exchanger ammonium molybdophosphate (AMP) has a great advantage over other materials in the separation and extraction of Cs^+^, and AMP was found as a microcrystalline powder that has a small specific surface area and poor mechanical strength. Within this context, porous and environmentally friendly polyurethane sponge (PU sponge) was selected as the carrier of AMP and ZrP. After physical impregnation, the self-polymerization and film-forming properties of dopamine at pH 8.5 were used to improve the storage of AMP and ZrP to prepare a novel cesium-adsorbed AMP- and ZrP-modified dual-channel composite adsorbent (AMP/PU and ZrP/PU sponges). The adsorption influence of AMP/PU and ZrP/PU sponges on Cs^+^ and the possible factors affecting the adsorption were investigated to explore the adsorption mechanism. This work serves as a novel method for the efficient adsorption of Cs^+^ in brine.

## 2. Experiments

### 2.1. Material and Characterization

The reagents were used without any further purification. Polyurethane sponge (PU sponge) was purchased from local supermarket. Ammonium molybdophosphate ((NH_4_)_3_PMo_12_O_40_·xH_2_O, AMP) and zirconium phosphate (Zr(HPO_4_)_2_·2H_2_O, ZrP) were provided by Aladdin analytical reagents. Dopamine hydrochloride (C_8_H_11_NO_2_·HCl) and tris-hydroxy-methy-lamino-methane (C_4_H_11_NO_3_) were purchased from Fuyu Fine Chemical Co., Ltd. (Tianjin, China). Cesium chloride (CsCl), sodium hydroxide (NaOH), and hydrochloric acid (HCl) were provided by Aladdin analytical reagents. And the CsCl stock solution was obtained by dissolving CsCl in ultrapure water.

The structure of AMP/PU and ZrP/PU sponges was analyzed using the transform infrared spectroscopy (Magna560, FT-IR) in the range of 400–4000 cm^−1^. The thermal stability of materials was determined using the thermal analysis instrument (NETZSCA-STA, DSC), ranging from 25 to 800 °C with a heating ramp of 10 °C under a nitrogen gas environment. The morphology of the adsorbents was characterized using the field emission scanning electron microscope (JSM-7900F, SEM, Jeol Ltd., Tokyo, Japan). The standard addition method commonly used by an atom adsorption spectrometry (ICE-3000, AAS, Thermo Fisher Scientific Inc., Waltham, MA, USA) determined the mass concentration of Cs^+^ in the solution before and after adsorption. The specific method is as follows: first, take several equal portions of the sample solution (solution to be tested); second, add standard solutions with different concentrations; then, measure the adsorbance and draw the adsorbance curve; finally, extrapolate the original Cs^+^ concentration from the adsorption curve. 

### 2.2. Preparation of AMP/PU and ZrP/PU Sponge

The PU sponge (1 × 1 × 1 cm^3^) was soaked in 1 mol/L NaOH to remove the oil from the surface, and then washed with deionized water. Later on, the PU sponge was immersed in a solution of 0.3 g AMP dispersed with 50 mL ultrapure water, until it was fully adsorbed and dried in a vacuum (adsorption, drying steps are repeated until the PU sponge completely sucks up the AMP dispersion). Then, tris reagent was used as a buffer to adjust the 2 mg/mL dopamine solution to 0.1 mol/L, and the pH was adjusted to 8.5 with 0.1 mol/L HCl. Finally, the PU sponge in the previous step was soaked in dopamine buffer at room temperature for 8 h; after, it was washed with ultrapure water and dried to obtain AMP/PU sponge. The preparation method and conditions of the ZrP/PU sponge were similar to those of 2.2, and only AMP was replaced with ZrP.

### 2.3. Adsorption Experiments

Batch adsorption tests were conducted to determine the influence of the adsorbent dosage (0–0.05 g), solution pH (1.0–12.0), temperature (293–338 K), initial concentration (5–35 mg/L), contact time (0.17–12 h), adsorption kinetics, adsorption isotherm, as well as adsorption thermodynamics on the adsorption performance. 

The experimental details are as follows: the 1 × 1 × 1 cm^3^ sponge adsorbent (the mass of ZrP and AMP is 0.025 g, excluding dosage experiment) was added to the 15 mL, 25 mg/L Cs^+^ solution (excluding initial concentration experiment) at pH 7.0 (excluding pH experiment), and the adsorption experiment of Cs^+^ was carried out at 298 K (excluding temperature experiment) with stirring (120 r/min) for 2 h (excluding contact time experiment). The optimal adsorption material and adsorption conditions were selected by calculating adsorption performance parameters.

(1) $$Qe-\left(C_{0}-C_{e}\right)V/m$$

(2) $$AE=(C_{0}-C_{e})/C_{0}\times 100\%$$

where Q_e_ is the equilibrium adsorption capacity (mg/g), V is the volume of the solution (mL), C_0_ is the initial concentration of the solution before adsorption (mg/L), C_e_ is the concentration of the solution after adsorption equilibrium (mg/L), m is the amount of adsorbent (g), and AE is the equilibrium adsorption efficiency (%).

### 2.4. Reusability Experiments

The HCl (0.5 mol/L) was eluted by continuously stirring with the speed of 600 r/min for 1 h at 293 K; after, the adsorbents were saturated via adsorption. Then, solid–liquid separation was performed to measure the Cs^+^ concentration using AAS to calculate the desorption efficiency. The above processes were cycled to evaluate the reusability and stability of AMP/PU and ZrP/PU sponges.

### 2.5. DFT Calculations

Ammonium phosphomolybdate (AMP) and zirconium phosphate (ZrP) were selected as adsorbents for Cs^+^ adsorption, and porous materials were used as matrix carriers of inorganic adsorption materials. AMP and ZrP were used as adsorbents to study the adsorption mechanism of composite adsorbents for Cs^+^ to simplify the description. All the calculations were performed within the framework of the density functional theory (DFT) as implemented in the Vienna Ab initio Software Package (VASP 5.3.5) code within the Perdew–Burke–Ernzerhof (PBE) generalized gradient approximation and the projected augmented wave (PAW) method [19]. The cutoff energy for the plane-wave basis set was set to 400 eV. The Brillouin zone of the surface unit cell was sampled using Monkhorst-Pack (MP) grids, with a k-point mesh density of 2π × 0.04 Å^−1^ for structure optimization [20]. The convergence criteria for the electronic self-consistent iteration and force were set to 10^−5^ eV and 0.01 eV/Å, respectively. 

## 3. Results and Discussion

### 3.1. Characterization of AMP/PU and ZrP/PU Sponges

**Figure 1:**
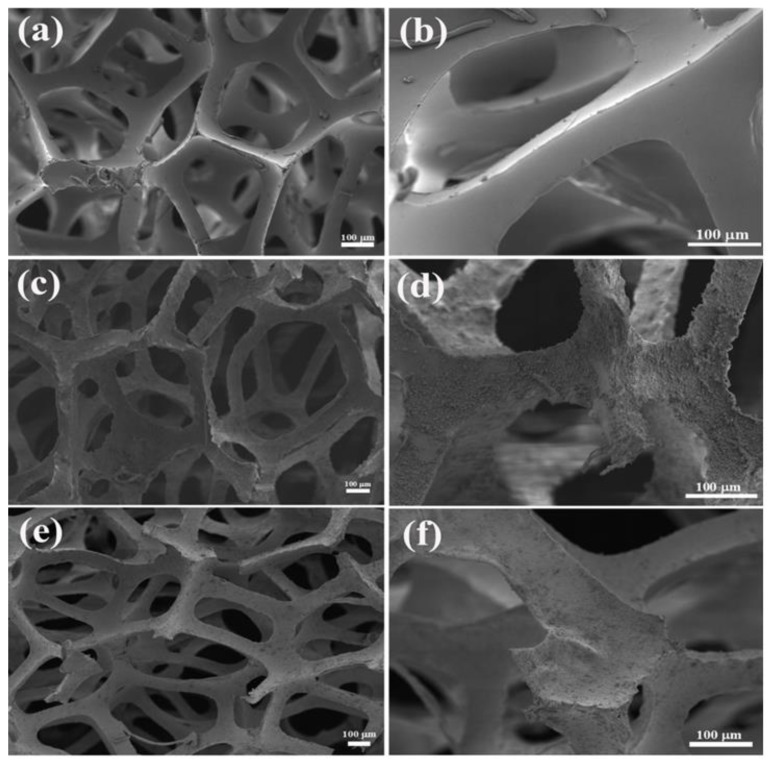
SEM of PU sponge (**a**,**b**), AMP/PU sponge (**c**,**d**), and ZrP/PU sponge (**e**,**f**).

The morphology of the adsorbents was revealed via SEM. It can be observed in Figure 1a,b that the PU sponge is a three-dimensional porous network structure with a smooth skeleton and micron pores. This uniformly distributed and highly ordered macroporous framework provides a large surface area and pores for the immobilization of ZrP and AMP, and achieves the benefit of a higher adsorption capacity. It can be seen from Figure 1c that the AMP/PU sponge inherits the network structure of the PU sponge interconnection, indicating that the sponge skeleton is not destroyed during the production process. But the surface of the AMP/PU sponge skeleton becomes rougher compared with the PU sponge. From the AMP/PU sponge detail shown in Figure 1d, it can be seen that the smooth PU sponge’s surface is uniformly attached to a dense and random AMP particle, which suggests that the particle was uniformly immobilized on the PU sponge. Similarly, the morphology of the ZrP/PU sponge shown in Figure 1e,f also suggests that ZrP is uniformly immobilized on the PU sponge’s surface.

**Figure 2:**
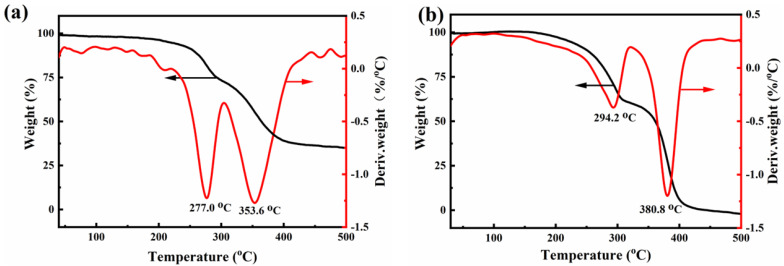
TG−DTG of AMP/PU sponge (**a**) and (**b**) ZrP/PU sponge.

The TG-DTG of the adsorbents are shown in Figure 2. For the thermogravimetric analysis, the protective gas used was nitrogen, and the test temperature was taken from 30.0 to 500.0 °C. As can be seen in Figure 2a, the first pyrolysis loss mass range of the AMP/PU sponge was from 258.0 to 288.8 °C, and the loss mass was about 22.39%. The main mass loss interval was from 328.5 to 384.9 °C, and the loss mass was about 34.1%, while the end solid residue was 34.63%. From Figure 2b, it can be seen that the ZrP/PU sponge had a two-stage mass loss; the first weight loss range was from 258.6 to 305.9 °C, and the loss mass was about 38.99%. The main loss mass range was from 363.2 to 396.6 °C, and the loss mass was about 59.27%, while the end solid residue was 2.48%. Thus, the quality of the AMP/PU and ZrP/PU sponges remains constant at 25–250 °C, indicating thermal stability within 250 °C. 

**Figure 3:**
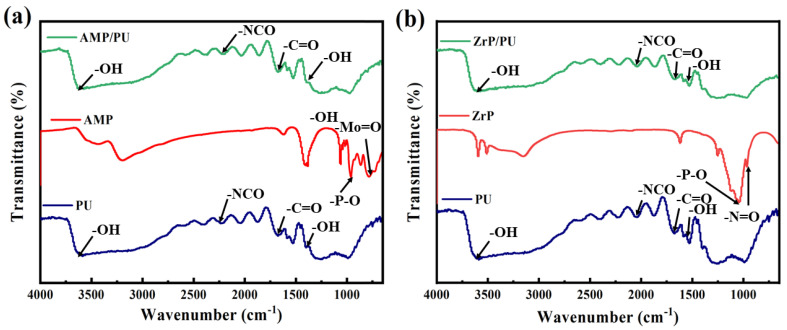
FT−IR of AMP/PU (**a**) and (**b**) ZrP/PU sponges and the corresponding precursors.

The chemical structure of the sponge adsorbent was analyzed via FT-IR. As shown in Figure 3, the infrared spectra of the original PU, AMP/PU, and ZrP/PU show minimal differences. Because the preparation process belongs to the physical changes and does not involve changes in the chemical structure, the composite AMP/PU and ZrP/PU spectra are similar to the original PU, but this does not mean that there is no change at all. The characteristic peak of the free NH is approximately 3400 cm^−1^. Owing to the action of the hydrogen bond, the characteristic peak of the NH will shift to the lower position. There is a strong adsorption peak at 3315 cm^−1^, and the peak type is not very sharp. This peak is the stretching vibration adsorption peak of NH, which is the characteristic adsorption peak of the NH group in polyurethane. The stretching vibration of C=O is present in the range of 1850–1660 cm^−1^, and the adsorption peak near 1719 cm^−1^ is the stretching vibration adsorption peak of C=O in carbamate [21]. From Figure 3a, it can be seen that the four vibrational bands at 1062, 964, 862, and 774 cm^−1^ show the existence of P-O, Mo-O, and Mo-O-Mo groups, which are attributable to the intact Keggin structure of [PMo_12_O_40_]^3-^, and the AMP/PU sponge was successfully obtained [16]. As shown in Figure 3b, the peaks at 970 cm^−1^ and 1251 cm^−1^ belong to the out-of-plane vibration and in-plane vibration of the P-OH group, respectively [22]. The peak at 593 cm^−1^ is attributed to the vibration of Zr-O. The symmetry and bending vibrations of the -OH groups from the ZrP lattice water were brought by the spectra at 3465 cm^−1^ and 1672 cm^−1^, and the ZrP/PU sponge was successfully obtained [23].

### 3.2. Effect of Dosage

**Figure 4:**
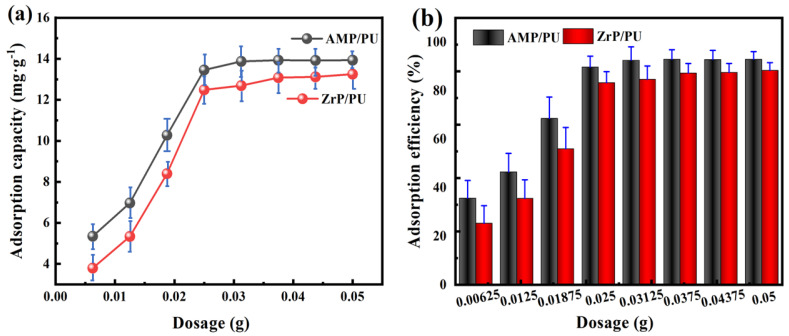
Effect of dosage on adsorption capacity (**a**) and adsorption efficiency (**b**) of Cs^+^.

The effect of the dosage of adsorbents on the adsorption performance of Cs^+^ was studied. The results are shown in Figure 4; the adsorption performance of the AMP/PU sponge is higher than that of the ZrP/PU sponge. Figure 4a shows that the adsorption capacity gradually increases as the adsorbent dosage increases. The adsorption capacity reaches saturation when the adsorbent dosage reaches 0.025 g. In Figure 4b, it can be seen that the adsorption efficiency increases with the increase in the adsorbent dosage, and the adsorption efficiency tends to become balanced when the dosage reaches 0.025 g. This is because with the increase in the dosage of adsorbents, there are more adsorption sites available for Cs^+^ adsorption, and the existing Cs^+^ is adsorbed; continuing to increase the dosage cannot significantly improve the adsorption.

### 3.3. Effect of Solution pH

**Figure 5:**
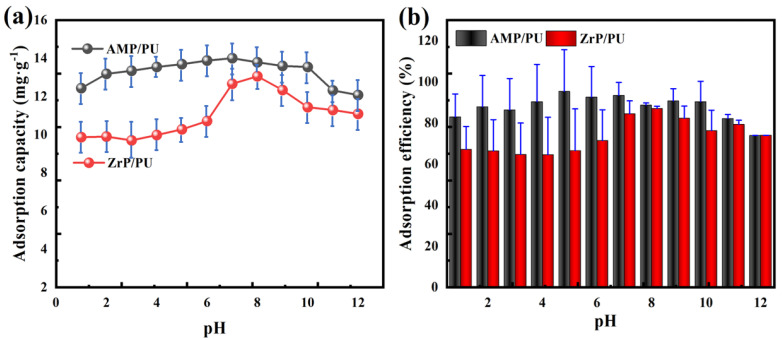
Effect of pH on adsorption capacity (**a**) and adsorption efficiency (**b**) of Cs^+^.

The pH of the solution affects the surface charge of the solid and further affects the adsorption behavior. Humic acid (HA) in water may interact with the adsorbent through hydroxyl, carbon-based, and carboxyl groups, and may affect the adsorption of cesium [7,24,25]. The pH was adjusted using HCl and NaOH for the analysis and study. The results in Figure 5 show that the adsorption performance of the AMP/PU sponge is higher than that of the ZrP/PU sponge. From Figure 5a, it can be seen that the adsorption capacity of the AMP/PU sponge has a slight increase at pH < 10, which is due to the AMP’s acid resistance [26]. When pH > 10, under alkaline conditions, the AMP reacts with OH^−^ [27] as follows:

$${\left({NH}_{4}\right)}_{3}PO_{4}\cdot 12MoO_{3}+27OH^{-}\to 3{NH}_{3}\cdot H_{2}O+PO_{4}^{3-}+12MoO_{4}^{2-}+12H_{2}O$$

which causes the structure of AMP to be destroyed, leading to the decrease in the adsorption capacity of AMP/PU. Correspondingly, at a pH < 6, the adsorption capacity of the ZrP/PU sponge increases slightly, which is caused by a greater amount of H^+^, which competes with the Cs^+^. The adsorption capacity of the ZrP/PU sponge increases significantly at a pH of 6–9; this is because the solution becomes neutral, and the H^+^ that competes with Cs^+^ decreases. When the pH > 9, the Cs^+^ is hydrolyzed to form more alkaline hydroxide cesium, resulting in a decrease in the capacity of the Cs^+^ adsorbed. Similarly, in Figure 5b, the adsorption efficiency shows the same change as the adsorption capacity.

### 3.4. Contact Time and Kinetics Studies

**Figure 6:**
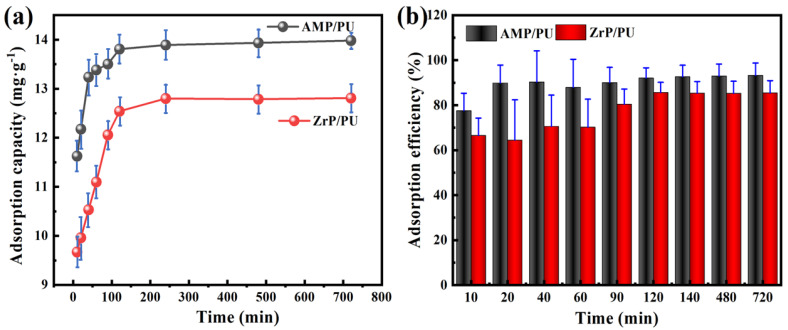
Effect of contact time on adsorption capacity (**a**) and adsorption efficiency (**b**) of Cs(I) ions.

The Cs^+^ adsorption kinetics is presented in Figure 6. Initially, the adsorbents’ adsorption was relatively fast, and the process slowed down afterward, attaining equilibrium at about 2 h. It was found that the adsorption performance of the AMP/PU sponge was better than that of the ZrP/PU sponge because there were a lot of adsorption sites on the surfaces of the AMP/PU and ZrP/PU sponges at the beginning. As time goes by, the adsorbents adsorb a considerable amount of Cs^+^, and the adsorption sites decrease, which hinders the adsorption of Cs^+^ and tends to balance the capacity. The adsorption efficiency in Figure 6b shows a similar regulation to Figure 6a.

Studying the kinetics can help to infer the reaction mechanism. The adsorption kinetic parameters were obtained by estimating the adsorption rate and the expression in the adsorption process, and by establishing an appropriate kinetic model to simulate the experimental process [7]. The kinetic equation (Table 1) was used to fit the dynamic data. When the fitting coefficient R^2^ is closer to 1, this indicates that the adsorption process is fitting to the kinetic model, and the relationship between the adsorption efficiency and the contact time in the solid–liquid adsorption process are then studied [28]. We estimated the experimental results (Qe) based on the kinetic equation, obtained the corresponding adsorption kinetic parameters and data (Qt), and then established an accurate and suitable model to simulate and analyze the process.

Q_e_ and Q_t_ are the adsorption capacities at equilibrium (mg/g) and time t (mg/g), respectively, K_1_ is the rate parameter of the first-order kinetics, K_2_ is the rate parameter of the second-order kinetics, K_3_ is the rate parameter of the particle diffusion kinetics, and C represents the constant of the particle diffusion kinetic model.

**Figure 7:**
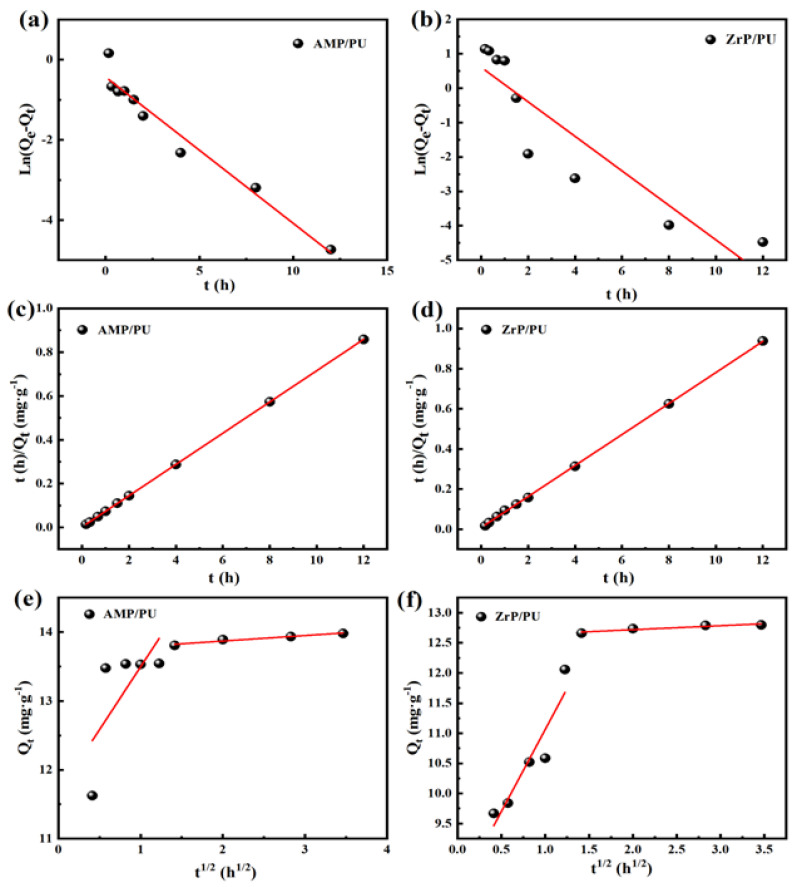
Kinetic model fitting of adsorbent for Cs^+^: (**a**,**b**) quasi-first-order kinetics, (**c**,**d**) quasi-second-order kinetics, and (**e**,**f**) particle diffusion kinetics.

The fitting results of the kinetics are found in Figure 7 and Table 2, which show that the fitting coefficient of the quasi-second-order kinetics to the AMP/PU and ZrP/PU sponges’ adsorption of Cs^+^ is closer to 1 than the other models (AMP/PU sponge, R^2^ = 0.9998; ZrP/PU sponge, R^2^ = 0.9997), indicating that the reaction process is greatly affected by the adsorption site. At the same time, this shows that the Cs^+^ adsorption in this stage is mainly controlled by the diffusion within the particles and chemical adsorption [29].

### 3.5. Effect of Cs^+^ Initial Concentration and Adsorption Isotherms

**Figure 8:**
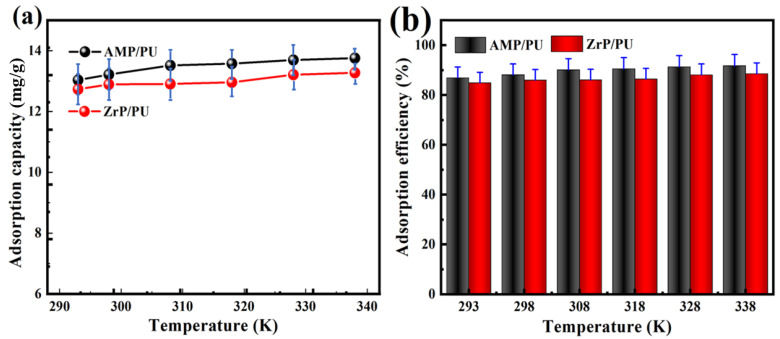
Effect of initial Cs^+^ concentration on adsorption capacity (**a**) and adsorption efficiency (**b**) of Cs^+^.

In Figure 8a, it is seen that when the Cs^+^ concentration is 5–35 mg/L, the adsorption capacity of the adsorbent increases significantly with the increase in the initial concentration of Cs^+^, which is due to the gradual increase in the Cs^+^, and the adsorbents can bind to the adsorption of Cs^+^. The adsorption efficiency of the adsorbent, shown in Figure 8b, remains above 80%, indicating that the adsorption efficiency of the adsorbent remains stable at 5–35 mg/L. Moreover, the results show that the AMP/PU sponge adsorption performance is better than that of the ZrP/PU sponge.

The adsorption isotherm is the relationship between the adsorption capacity and the equilibrium concentration when the adsorption system reaches equilibrium at a certain temperature. Characteristic parameters describing the adsorption process (such as adsorption capacity) can provide theoretical data for the study of the adsorption mechanism and thermodynamics [7]. The adsorption data are fitted by the adsorption model (Table 3); when the fitting coefficient R^2^ is closer to 1, this indicates that the adsorption process conforms to the hypothetical theory of the model, which accurately describes the adsorption mechanism between the adsorbent and the Cs^+^.

C_e_ is the concentration at the adsorption equilibrium (mg/L), Q_e_ is the adsorption capacity under the adsorption equilibrium (mg/g), Q_m_ is the saturated adsorption capacity (mg/g), K_L_ is the Langmuir adsorption equilibrium constant, R_L_ is the separation factor parameter, and K_F_ is the adsorption capacity of the adsorbent for adsorbed ions. The adsorption index is represented by 1/n, and the smaller the 1/n is, the better the adsorption capacity; A and B are the Temkin constants (L/mg).

**Figure 9:**
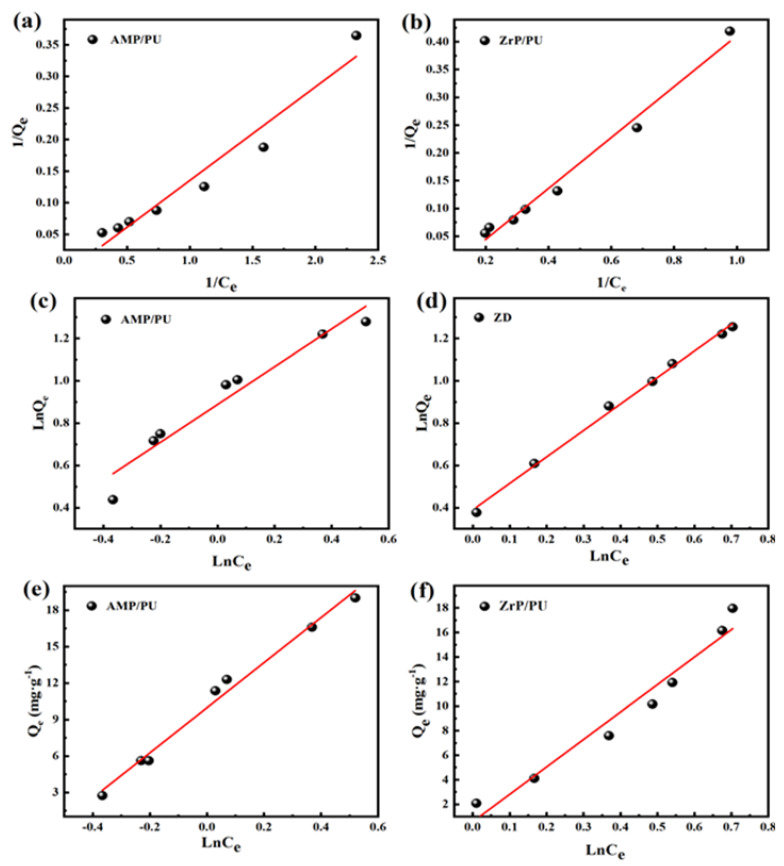
Isothermal adsorption model fitting of Cs^+^ on adsorbent: (**a**,**b**) Langmuir, (**c**,**d**) Freundlich, and (**e**,**f**) Temkin.

Figure 9 and Table 4 are the fitting results of the adsorbent isothermal adsorption. It is apparent that the adsorption of the AMP/PU sponge is well fitted to the Temkin model (R^2^ = 0.985), which indicates that the surface of the AMP/PU sponge is uniformly adsorbed. The ZrP/PU sponge adsorption of Cs^+^ is linear with the Freundlich model (R^2^ = 0.995), indicating that the adsorption process is relatively difficult (1/n = 1.250, if 1/n > 2 considered difficult to adsorb). The Dubinin–Radushkevich isotherm model is used in [30].

### 3.6. Effect of Temperature and Thermodynamics

**Figure 10:**
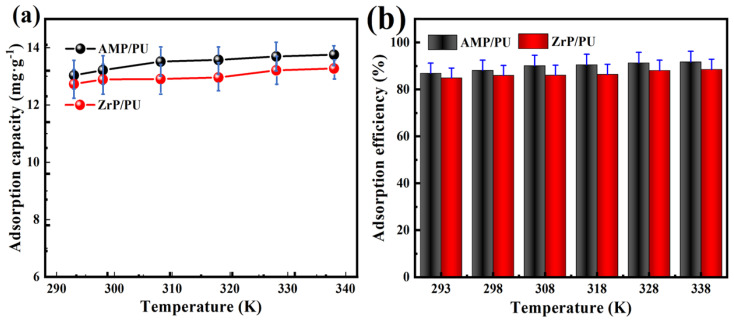
Effect of temperature on adsorption capacity (**a**) and adsorption efficiency (**b**) of Cs^+^.

The changes in the Cs^+^ adsorption performance with the temperature were studied. As shown in Figure 10, the adsorption performance of the AMP/PU sponge to the Cs^+^ is better than that of the ZrP/PU sponge. As shown in Figure 10a, the adsorption process appears as a slower capture stage as the temperature increases; the Cs^+^ adsorption capacity is higher at a higher temperature, which may be due to the increased random thermal movement of Cs^+^ at high temperatures, which increases the possibility of collisions between the Cs^+^ and the binding site of the adsorbent. This indicates that the adsorption of Cs^+^ by the adsorbent is an endothermic process. At the same time, due to the limited binding sites, the fluctuation range of the adsorption capacity is small with the increase in the temperature. Figure 10b shows a similar change to Figure 10a; the adsorption efficiency of the adsorbent remains above 82%, which shows that the AMP/PU and ZrP/PU sponges have thermal stability and an excellent adsorption performance for Cs^+^.

The adsorption process goes hand in hand with the change in the system energy. The thermodynamic parameters are the basic indicators to judge the spontaneity of the adsorption reaction process, including Gibbs free energy (ΔG), enthalpy (ΔH), and entropy (ΔS). The thermodynamic parameters play important roles in the energy change, in the adsorption process, and in determining whether the adsorption process can proceed spontaneously [31]. The thermodynamic parameters and the spontaneous calculation of the Cs^+^ adsorption are determined by analyzing the thermodynamic experiments, and the formula is as follows:

(3) $$K_{d}=Q_{e}/C_{e}$$

(4) $$∆G=-RTLnK_{d}$$

(5) $$LnK_{d}=∆S/R-∆H/RT$$

where Q_e_—the adsorption capacity at the adsorption equilibrium (mg/g); C_e_—the concentration at the adsorption equilibrium (mg/L); K_d_—the distribution coefficient during the adsorption process; R—the gas constant (8.314 J/mol·K); T—the absolute temperature during adsorption (K); ΔH—the enthalpy of the adsorption isotherm (kJ/mol); and ΔG—Gibbs free energy (J/mol/K).

**Figure 11:**
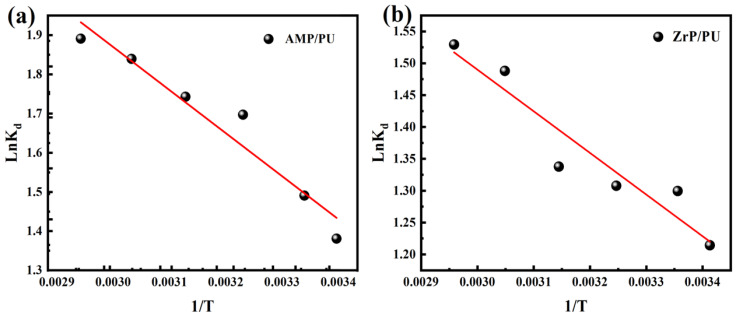
Van’t Hoff plot of adsorbents regarding the adsorption of Cs^+^: (**a**) AMP/PU and (**b**) ZrP/PU.

The van’t Hoff and thermodynamic parameters are shown in Table 5, and the van’t Hoff, shown in Figure 11, is found to be fine linear. The negative ΔG at different temperatures verify the spontaneity and feasibility of the adsorption, and indicate that the adsorption capacity of the adsorbent for Cs^+^ has a higher driving force at high temperatures [32]. A positive value of ΔH indicates that the adsorption process is endothermic. In addition, the change in the entropy (ΔS) reflects the net effect of the increase in the surface change of the adsorbent (ΔS1) and the loss of freedom of the adsorbent (ΔS2). The positive entropy proves that ΔS1 exceeds ΔS2 in value, indicating that the degree of freedom and randomness increase during the adsorption process.

### 3.7. Mechanism of Adsorption

**Figure 12:**
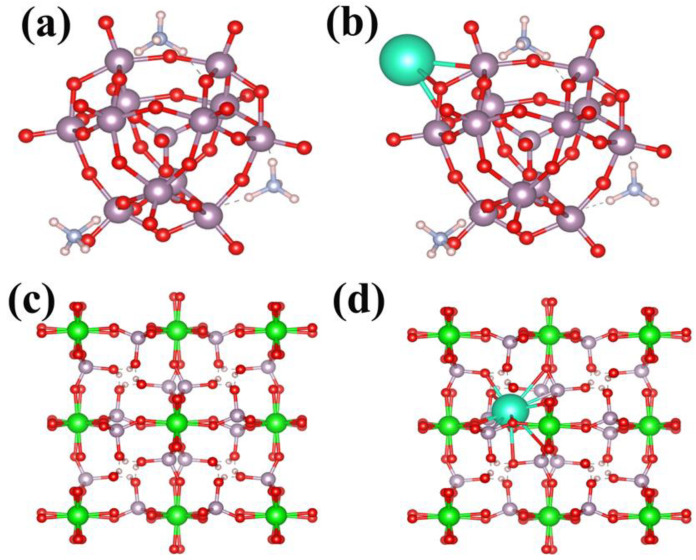
DFT calculation before and after Cs^+^ adsorption: (**a**) AMP, (**b**) AMP after adsorbing Cs^+^, (**c**) ZrP, and (**d**) ZrP after adsorbing Cs^+^.

According to previous studies, the molecular hybridization between inorganic adsorption materials and loaded carriers is usually achieved through electrostatic interactions. This method is simple and direct, but this type of adsorption material is prone to the phenomenon of inorganic ion exchange agent detachment during use, with poor stability and a low adsorption capacity, and an inconvenient enrichment and recovery. Therefore, the use of covalent bond hybridization may solve the problem of the inorganic ion exchanger easily falling off in the process of adsorbing materials, as shown in Figure 12a,b; the AMP/PU sponge prepared and synthesized in this work undergoes ion exchange between the Cs^+^ and NH_4_^+^ in the AMP on the AMP/PU sponge.

(NH_4_) _3_PMo_12_O_40_·xH_2_O+3Cs^+^ → Cs_3_PMo_12_O_40_ xH_2_O + 3NH_4_Cl selectively adsorbs Cs^+^, demonstrating the characteristics of chemical adsorption. This adsorption method has a strong adhesion and specificity. On the contrary, the ZrP pairs in the ZrP/PU sponges adsorb Cs^+^ through electrostatic attraction, which belongs to physical adsorption. The Cs^+^ forms P-O-M (M: metal ions) through electrostatic attraction and is adsorbed on the ZrP/PU, as shown in Figure 12c,d.

The AMP/PU and ZrP/PU sponges’ Cs^+^ adsorbents show different adsorption mechanisms. The former forms a covalent bond through the exchange of Cs^+^ and NH_4_^+^ in the AMP, which belongs to chemical adsorption. Therefore, the adsorbent has a strong selectivity and excellent fantasy stability. The latter physically adsorbs Cs^+^ through electrostatic action. However, the adsorption capacity of both for Cs^+^ decreases with time due to the occupation of adsorption sites as the adsorption site progresses. Therefore, a 3D porous adsorbent with a high specific surface area and a high active site is better for Cs^+^ adsorption.

## 4. Conclusions

In short, by using ammonium molybdate (AMP) and zirconium phosphate (ZrP) as raw materials and a low-cost porous polyurethane (PU) sponge as a carrier, two dual-channel adsorbents were synthesized via physical impregnation and dopamine sealing, and the effective adsorption of Cs^+^ in an aqueous solution was achieved. Both adsorbents have a good thermal stability within 250 °C. When the mass of the adsorbent is 0.025 g (volume is 1 × 1 × 1 cm^3^) and the concentration of Cs+ is within the range of 5–35 mg/L, the optimal adsorption effect is achieved when the pH value of the solution is 7.0. AMP/PU, and the ZrP/PU reaches adsorption equilibrium within 2 h. The adsorption rate can reach around 90%. Under the same conditions, the adsorption effect of the AMP/PU on the Cs^+^ is better than that of the ZrP/PU. Through adsorption kinetics research, it was found that the adsorption processes of the AMP/PU and ZrP/PU follow a quasi-second-order kinetics model, and the adsorption process is called chemical adsorption. The equilibrium adsorption capacities are 14.0075 mg/g and 12.9416 mg/g, respectively. The isothermal adsorption line study shows that the adsorption of Cs^+^ by the AMP/PU is more in line with the Temkin model, and the surface of the adsorbent is uniformly adsorbed. The adsorption of Cs^+^ by the ZrP/PU is more in line with the Freundlich model, and the adsorption process for the Cs^+^ is mainly multi-layered. The thermodynamic study shows that the adsorption process is a spontaneous endothermic process. The mechanism of the selective adsorption of Cs^+^ by the AMP/PU is the ion exchange between the Cs^+^ and NH_4_^+^ in the gap of the AMP structure. The mechanism of the selective adsorption of Cs^+^ by the ZrP/PU is that ZrP adsorbs Cs^+^ through electrostatic attraction, which forms P-O-M (M: metal ions) for adsorption.

This adsorbent has certain advantages such as stability and regeneration, low cost, simple process, and environmental friendliness, and has the potential to be applied in the fields of low-concentration brine extraction of Cs^+^ and industrial wastewater treatment. However, in general, AMP and ZrP are dispersible and hydrophilic, often appearing in the form of ultrafine powders in water, which may hinder their separation from water after being used as Cs^+^ adsorbents, thus limiting their use in water treatment.

## Figures and Tables

**Table 1:** Adsorption kinetics model [7].

Kinetic Equations	Formula	Theory
Quasi-first-order dynamic model	$Ln(Q_{e}-Q_{t})=LnQ_{e}-K_{1}t$	The adsorption process is controlled by the diffusion step. The adsorption efficiency is proportional to the difference between the equilibrium adsorption capacity and the adsorption capacity at a certain time.
Quasi-second-order dynamic model	$t/Q_{t}=1/(K_{2}Q_{e}^{2})+t/Q_{e}$	The adsorption efficiency is controlled by the chemical adsorption mechanism. The adsorption process involves electron sharing or electron transfer between the adsorbent and Cs^+^.
Particle diffusion dynamics model	$Q_{t}=K_{3}t^{1/2}+C$	The adsorption process is controlled by a variety of diffusion mechanisms, including the diffusion of Cs^+^ inside or outside the adsorbent.

**Table 2:** Different kinetic model fitting parameters and determination coefficients of adsorbents for Cs^+^.

Kinetic Equations	Adsorbents (Sponges)	Fitting Parameters		Determination Coefficients
		K_1_ (h^−1^)	Q_e_ (mg/g)	R^2^
Quasi-first-order dynamic model	AMP/PU	0.364	0.647	0.955
	ZrP/PU	0.502	0.543	0.813
		K_2_ (g/mg·h^−1^)	Q_e_ (mg/g)	R^2^
Quasi-second-order dynamic model	AMP/PU	2.255	14.007	0.999
	ZrP/PU	0.705	12.941	0.999
		K_3_ (mg/g·h^0.5^)	C	R^2^
Particle diffusion dynamics model I	AMP/PU	1.823	11.673	0.314
	ZrP/PU	2.723	8.340	0.841
Particle diffusion dynamics model II	AMP/PU	0.079	13.710	0.931
	ZrP/PU	0.066	12.585	0.842

**Table 3:** Isothermal adsorption model.

Adsorption Isotherm	Formula	Constant	Theory
Langmuir	$\frac{1}{Q_{e}}=\frac{1}{{K_{L}Q}_{m}C_{e}}+\frac{1}{Q_{m}}$ $R_{L}=1/(1+K_{L}C_{0})$	R_L_ = 0, irreversible adsorption. 0 < R_L_ < 1, favorable adsorption. R_L_ = 1, linear adsorption. R_L_ > 1, adverse adsorption.	The adsorption of Cs^+^ on the surface of the adsorbent is single-layer adsorption. The adsorption site is uniform, and the adsorption is located. There is no interaction between the adsorbed ions. The adsorption capacity is unchanged after the adsorbent reaches saturation.
Freundlich	$LnQ_{e}=LnK_{F}+(LnC_{e})/n$	The smaller the 1/n is, the better the adsorption. 0 < 1/n < 1 is easy to adsorb. 1/n > 2 is difficult to adsorb.	The adsorption process is considered to be multi-layer adsorption. The adsorption sites on the surface of the adsorbent are not uniform. The adsorption sites are different.
Temkin	$Q_{e}=BLnA+BLnC_{e}$	A is related to heat of adsorption.	The interaction between the adsorbed ions in the adsorption process will affect the adsorption equilibrium to some extent (the decrease in the intermolecular heat of adsorption). The adsorbent is uniform on the adsorbent surface.

**Table 4:** Different isothermal adsorption model fitting parameters and determination coefficients of adsorbents for Cs^+^.

Isothermal Adsorption Model	Adsorbents (Sponges)	Fitting Parameters		Determination Coefficients
		K_L_ (L/g)	Q_m_ (mg/g)	R^2^
Langmuir	AMP/PU	0.085	78.678	0.942
	ZrP/PU	0.102	21.244	0.983
		K_F_ (mg/g)	1/n	R^2^
Freundlich	AMP/PU	2.430	0.891	0.931
	ZrP/PU	1.477	1.250	0.995
		A (L/mg)	B (L/mg)	R^2^
Temkin	AMP/PU	1.711	18.554	0.985
	ZrP/PU	1.026	22.365	0.951

**Table 5:** Adsorption thermodynamic parameters of adsorbents for Cs^+^.

Temperature (K)	−ΔG (kJ/mol)		ΔH (kJ/mol)	
	AMP/PU Sponge	ZrP/PU Sponge		
293	3.364	2.958	AMP/PU sponge	ZrP/PU sponge
298	3.693	3.219	9.127	5.432
308	4.345	3.348	ΔS(J/mol/K)	
318	4.607	3.536		
328	5.016	4.057	AMP/PU sponge	ZrP/PU sponge
338	5.313	4.297	43.074	28.685

## Data Availability

We guarantee the authenticity, reliability, and effectiveness of the data.
